# Guns in the heartland: examining firearm behaviors among Oklahoman adults

**DOI:** 10.1016/j.pmedr.2026.103673

**Published:** 2026-10-18

**Authors:** Hillary McGuire, Marshall K. Cheney, Daniel Larson, Hairong Song, Jeff R. Temple, Yu Lu

**Affiliations:** a Department of Health and Exercise Science, University of Oklahoma, Norman, OK 73019, USA; b Department of Psychology, University of Oklahoma, Norman, OK 73019, USA; c School of Behavioral Health Sciences, The University of Texas Health Science Center at Houston, Houston, TX 77225, USA

**Keywords:** Firearms, Firearm storage, Firearm carrying, Oklahoma, Adults

## Abstract

**Objective:**

To assess firearm ownership, storage, carrying, and exposure to firearm-related threats among adults in Oklahoma, a state with high firearm-related mortality and permissive firearm laws.

**Methods:**

We analyzed cross-sectional data from the 2024 Oklahoma Firearm Survey, a community sample of 630 Oklahoma adults. We examined frequencies and percentages for each variable. We used χ^2^ tests to assess differences in firearm ownership, storage, carrying, and firearm-related threats by demographic characteristics, safety training, and concealed-carry permit status.

**Results:**

Overall, 51% of participants reported owning a firearm, and more than 30% reported carrying a firearm outside the home, most commonly for self-protection. Twenty-five percent reported having been threatened with a firearm. Threat exposure was more common among males, Black adults, individuals with less than a college education, and those employed full-time. Firearm owners were more likely to be male, live in rural areas, and be employed full-time. Nearly half of firearm owners (48.3%) and firearm carriers (48.5%) reported never receiving formal firearm safety training, and 90% of firearm owners reported unsafe storage practices.

**Conclusions:**

Firearm ownership and carrying were common in this Oklahoma sample, while unsafe storage and limited safety training were widespread. Targeted interventions are needed to reduce firearm-related harm.

## 1 Introduction

With over 46,000 firearm-related deaths in the U.S. in 2023 (Centers for Disease Control and Prevention, 2025), firearm violence is a public health crisis. While studies on firearms have increased in recent years (Degli Esposti et al., 2022; Kruis et al., 2020; Lu et al., 2024), there remains an overall need for a better understanding of even basic behaviors, especially in specific regions with high gun ownership and permissive gun laws, such as the state of Oklahoma. Oklahoma consistently ranks in the top 15 in firearm-related deaths, with a rate of 19.8 per 100,000 people (Centers for Disease Control and Prevention, 2022a), and from 2010 to 2020, the rate of firearm-related deaths increased by 25% (Centers for Disease Control and Prevention, 2022b). Despite these alarming statistics, little is known about Oklahomans' firearm behaviors. Existing national surveillance estimates indicate that 39% of Oklahomans reported having a firearm in the home, 41% kept it loaded, and 57% left loaded firearms unlocked (Friar et al., 2024). Although these estimates provide information about firearm presence and storage within the home, they overlook other critical firearm behaviors and experiences. The present study provides a comprehensive, Oklahoma-specific profile of firearm-related behaviors and experiences. Specifically, we assessed firearm ownership, storage practices, carriage, and lifetime exposure to firearm threats, and examined variation by demographic characteristics, safety training, and concealed-carry permit status. By providing detailed, state-specific data across multiple firearm behaviors and experiences, these findings can inform targeted public health interventions in a region with elevated firearm-related harm.

## 2 Methods

### 2.1 Study design and population

In the 2024 Oklahoma Firearm Survey (OFS), we administered an online survey to a community sample of Oklahoma adult residents (i.e., those aged 18+ who had lived in Oklahoma for 1+ years) about their firearm experiences. We employed recruitment methods that included digital channels, physical flyers distributed across Oklahoma, in-person outreach at community events, and word of mouth. Participants accessed the online Qualtrics® survey from January to June 2024. We had 739 non-fraudulent (i.e., not false respondents to gain incentives) individuals complete the survey; 109 (14.7%) participants were removed due to failing attention checks (*n* = 103) or for completing the survey in under 5 min (*n* = 6). The 13.9% attention-check exclusion rate falls within the 10.9%–35.7% range reported across online survey platforms (Gittner et al., 2026). The final sample yielded 630, representing 42 of Oklahoma's 77 counties. Participants were compensated with a $10 Amazon® gift card.

### 2.2 Measures

Participants reported the following firearm behaviors and experiences: ownership, storage, carrying, safety training, concealed-carry permit, and exposure to firearm violence. We assessed ownership with a yes/no question (“Do you personally own a working gun?”). We used three survey items to determine storage (“Where do you usually store your ammunition (ammo)?” “Do you store any of the guns you own in or around your home …” “…loaded?” “…unlocked?”), and filtered respondents into two groups: safe vs. unsafe storage. To adhere to safe firearm storage recommendations (American Academy of Pediatrics, 2012), we classified participants as ‘safe’ if they reported storing ammunition in a locked container, separate from firearms, and keeping firearms unloaded and locked; all other storage practices were categorized as ‘unsafe.’ We dichotomized carrying as ‘ever’ vs. ‘never carried’ a firearm outside the home in the past year (“Within the past 12 months, how often would you say you've carried a gun when you were outside your home – including in your car?”). Safety training (“Have you ever taken a firearm course from a certified firearm instructor?”) included three response options: “No,” “Yes, within the past 3 years,” and “Yes, but more than 3 years ago.” We coded responses as ‘never received training’ vs. ‘ever received training.’ For concealed-carry permit (“Have you ever had a concealed carry license?”), we coded responses as ‘never licensed’ vs. ‘ever licensed.’ We used five survey items to measure exposure to violent firearm use (“Have you ever been threatened with a gun?”, “Have you ever threatened someone else with a gun?” “Have you ever been shot with a gun?” “Have you ever shot someone else with a gun?” and “Have you ever shot yourself with a gun?”); the first two questions were answered with yes/no, and the latter three were answered with “no,” “yes, accidentally,” and “yes, intentionally.” We dichotomized all responses into yes vs. no.

We assessed standard sociodemographic characteristics, including age, gender, race/ethnicity, residential area, education, and past-year employment status. We calculated age as survey year minus reported birth year. We asked respondents to self-identify gender (male, female, transgender woman, transgender man, non-binary, other, or not sure); due to small cell sizes, we excluded non–male/female responses (*n* = 8) in the analysis. We coded race/ethnicity as non-Hispanic White, non-Hispanic Black, or other (Hispanic, American Indian/Alaska Native, Asian/Pacific Islander, or multiracial). We assessed residential areas as urban, suburban, or rural. We assessed educational attainment using seven response options and dichotomized education as ‘college and above’ (bachelor's or graduate degree) vs. ‘below college’ (all other education levels). We dichotomized past-year employment as ‘full-time’ (≥30 h/week) vs. ‘not full-time’ (part-time, out of work <1 year, out of work ≥1 year, retired, unable to work, or other [e.g., student or stay-at-home parent]).

### 2.3 Statistical analysis

We examined frequencies and percentages for each variable. We used χ^2^ tests to determine if firearm ownership, storage, carriage, and ever being threatened with a firearm differed by gender, race, education, residential area, and employment status. Fisher's Exact test was used when the expected cell size was smaller than 5. Other firearm violence exposure variables were not tested due to low endorsements. Additionally, we examined how firearm ownership, storage, and carrying differed by firearm safety training experience and concealed-carry permit status. Analyses were conducted using IBM SPSS Statistics®, version 28.0 (IBM Corporation, 2021).

We followed Institutional Review Board–approved consent, privacy, and safety protocols.

## 3 Results

Our community sample had a mean age of 36.1 years (SD = 13.4). Nearly half of respondents identified as male (45.7%) and just over half identified as female (53.0%). Most respondents reported being non-Hispanic White (55.7%), followed by non-Hispanic Black/African American (23.3%); the remaining 20.9% identified as another race/ethnicity, including Hispanic. Approximately half of the sample reported living in suburban areas (49.2%), with 37.4% living in urban areas and 13.3% in rural areas. Most respondents reported a college education or higher (74.0%), while 26.0% reported less than a college degree. In terms of employment, 60.7% reported full-time employment in the past year, whereas 39.7% reported not being employed full-time.

A slight majority of survey respondents (50.6%) owned a personal firearm; of those, 90% did not store their firearms safely. Thirty-one-point-2 % of participants reported carrying a firearm outside their home in the past year (for non-hunting, target shooting, or job-related purposes), with self-protection listed as the primary reason (81.2%). Respondents most often reported carrying automatic or semiautomatic handguns (67.4%); 77.9% bought these firearms from a store or person, and 14.2% had a family member loan a firearm. Fewer than 20% of respondents ever had a concealed-carry permit, and 31.7% received safety training from a certified professional at some point in their lifetime. Over 25% of respondents reported being threatened by a firearm, 4.9% have threatened others, 3.8% reported being shot by someone else, 1.5% had shot someone else, and 0.9% had shot themselves.

As shown in Table 1, Table 2, larger proportions of males (vs. females, 69.8% vs. 34.7%, χ^2^ = 76.1, *p* < .001), individuals living in rural areas (vs. urban or suburban areas, 66.2% vs. 53.2%, 43.7%, χ^2^ = 13.6, *p* = .001), and those employed full-time (vs. not full-time, 59.4% vs. 36.1%, χ^2^ = 29.9, *p* < .001) were firearm owners. Approximately half of firearm owners received firearm safety training with a certified instructor (vs. non-owners, 51.7% vs. 11.4%, χ^2^ = 109.0, *p* < .001) and some obtained a concealed-carry permit (vs. non-owners, 36.6% vs. 2.1%, χ^2^ = 110.4, *p* < .001). Alarmingly, 48.3% of firearm owners had no formal safety training, and 63.4% had no concealed-carry permit.

**Table 1:** Differences in firearm ownership, storage, carrying, and firearm-related threat exposure by gender and race/ethnicity among adults in Oklahoma, United States, 2024.

	Gender				Race				
	Female	Male			White	Black	All other races		
	N (%)	N (%)	Phi	χ^2^	N (%)	N (%)	N (%)	Cramer's V	χ^2^
**Firearm ownership**			0.350	76.1 (*p* < .001)				0.087	4.72 (*p* = .094)
Yes	116 (34.7)	201 (69.8)			183 (52.1)	80 (54.4)	56 (42.4)		
No	218 (65.3)	87 (30.2)			168 (47.9)	67 (45.6)	76 (57.6)		
**Firearm Storage**			−0.006	0.013 (*p* = .911)				0.119	4.53 (*p* = .104)
Safe	12 (10.3)	20 (10.0)			19 (10.4)	4 (4.6)	9 (16.1)		
Unsafe	104 (89.7)	181 (90.0)			164 (89.6)	76 (95.0)	47 (83.9)		
**Firearm carrying**			0.286	50.0 (*p* < .001)				0.080	3.97 (*p* = .137)
Yes	62 (19.0)	129 (45.6)			101 (29.3)	54 (38.0)	38 (29.0)		
No	265 (81.0)	154 (54.4)			244 (70.7)	88 (62.0)	93 (71.0)		
**Threatened with a firearm**			0.289	50.6 (*p* < .001)				0.187	21.4 (*p* < .001)
Yes	44 (13.6)	109 (38.8)			67† (19.6)	56⁎, § (39.7)	32† (24.6)		
No	280 (86.4)	172 (61.2)			275† (80.4)	85⁎, § (60.3)	98† (75.4)		

*Note.* The overall analytic sample included 630 adults; firearm storage analyses were limited to firearm owners (*n* = 319). All other races include Hispanic, Asian/Pacific Islander, American Indian/Native Alaskan, and more than one race. Reported percentages are row percentages.

*P* values were obtained using Pearson chi-square tests or Fisher's exact tests, as appropriate.

⁎ Different from Whites at the *p* < .05 level based on Bonferroni post hoc analysis.

† Different from Black at the p < .05 level based on Bonferroni post hoc analysis.

§ Different from all other races at the p < .05 level based on Bonferroni post hoc analysis.

**Table 2:** Differences in firearm ownership, storage, carrying, and firearm-related threat exposure by residential area, education, and employment status among adults in Oklahoma, United States, 2024.

	Residential Area					Education				Employment Status			
	Urban	Suburban	Rural			Below college	College and above			Full-time	Not full-time		
	N (%)	N (%)	N (%)	Cramer's V	χ^2^/Fisher's Exact test	N (%)	N (%)	Phi	χ^2^	N (%)	N (%)	Phi	χ^2^
**Firearm ownership**				0.153	13.6 (*p* = .001)			−0.002	0.002 (*p* = .961)			−0.228	29.9 (*p* < .001)
Yes	115^†§^ (53.2)	124⁎, § (43.7)	51⁎, † (66.2)			75 (50.0)	215 (50.2)			208 (59.4)	82 (36.1)		
No	101^†§^ (46.8)	160⁎, § (56.3)	26⁎, † (33.8)			75 (50.0)	213 (49.8)			142 (40.6)	145 (63.9)		
**Firearm Storage**				0.074	1.60 (*p* = .406)			0.020	0.119 (*p* = .730)			0.002	0.001 (*p* = .971)
Safe	8 (7.0)	14 (11.3)	6 (11.8)			8 (10.7)	20 (9.3)			20 (9.6)	8 (9.8)		
Unsafe	107 (93.0)	110 (88.7)	45 (88.2)			67 (89.3)	195 (90.7)			188 (90.4)	74 (90.2)		
**Firearm carrying**				0.088	4.44 (*p* = .108)			0.027	0.414 (*p* = .520)			−0.225	29.2 (*p* < .001)
Yes	75 (34.7)	74 (26.1)	24 (31.2)			48 (32.0)	125 (29.2)			134 (38.3)	39 (17.2)		
No	141 (65.3)	210 (73.9)	53 (68.8)			102 (68.0)	303 (70.8)			216 (61.7)	188 (82.8)		
**Threatened with a firearm**				0.049	1.40 (*p* = .497)			0.107	6.67 (*p* = .010)			−0.194	21.7 (*p* < .001)
Yes	61 (28.2)	68 (23.9)	18 (23.4)			50 (33.3)	97 (22.7)			113 (32.3)	34 (15.0)		
No	155 (71.8)	216 (76.1)	59 (76.6)			100 (66.7)	331 (77.3)			237 (67.7)	193 (85.0)		

*Note.* The overall analytic sample included 630 adults; firearm storage analyses were limited to firearm owners (*n* = 319). Reported percentages are row percentages.

*P* values were obtained using Pearson chi-square tests or Fisher's exact tests, as appropriate.

⁎ Different from Urban at the *p* < .05 level based on Bonferroni post hoc analysis.

† Different from Suburban at the p < .05 level based on Bonferroni post hoc analysis.

§ Different from Rural at the p < .05 level based on Bonferroni post hoc analysis.

Among firearm owners (*n* = 319), firearm storage behaviors did not differ by gender (χ^2^ = 0.013, *p* = .911), race (χ^2^ = 4.53, *p* = .104), residential area (χ^2^ = 1.60, *p* = .406), education level (χ^2^ = 0.119, *p* = .730), employment status (χ^2^ = 0.001, *p* = .971), firearm safety training (χ^2^ = 1.96, *p* = .161) or concealed-carry permit history (χ^2^ = 0.520, *p* = .473).

More males (vs. females, 45.6% vs. 19.0%, χ^2^ = 50.0, *p* < .001) and those employed full-time (vs. not full-time, 38.3% vs. 17.2%, χ^2^ = 29.2, *p* < .001) have carried a firearm outside the home in the past year. Of participants who reported carrying a firearm outside the home in the past year, nearly half received firearm safety training (vs. non-carriers, 51.4% vs. 23.2%, χ^2^ = 45.2, *p* < .001), and some obtained a concealed-carry permit (vs. non-carriers, 42.3% vs. 9.6%, χ^2^ = 83.1, *p* < .001). Nonetheless, 48.5% of firearm carriers had no formal safety training, and 57.7% had no concealed-carry permit.

Those identifying as males (vs. females, 38.8% vs. 13.6%, χ^2^ = 50.6, *p* < .001), Black (vs. White or all other races, 39.7% vs. 19.6%, 24.6%, χ^2^ = 21.4, *p* < .001), having below a college education (vs. college education or above, 33.3% vs. 22.7%, χ^2^ = 6.67, *p* = .010), and employed full-time (vs. not full-time, 32.3% vs. 15.0%, χ^2^ = 21.7, *p* < .001) more frequently had been threatened by someone with a firearm.

## 4 Discussion

In the first study to exclusively examine multiple firearm behaviors in an Oklahoman sample, 51% reported firearm ownership, which is higher than the national average (41%) (Schaeffer, 2021) and previous state estimates of guns in the home (39%) (Friar et al., 2024). Our findings address a gap in national data by focusing on a region with high firearm ownership and permissive policies, providing critical insights into firearm storage. We found widespread unsafe storage practices, which are concerning in the context of Oklahoma's relatively high firearm-related mortality rates (Anglemyer et al., 2014; Monuteaux et al., 2019). Notably, nearly 18% of eligible individuals who accessed our survey declined to participate, compared to 12% in a national survey (Friar et al., 2024). These lower response rates may reflect more conservative political views (Burton et al., 2021) and concerns over Second Amendment rights, thus resulting in an underreporting of firearm behaviors.

Similar to national trends (Kaufman et al., 2024) and gendered firearm norms (McDermott et al., 2021; Parker et al., 2017), Oklahoma firearm owners and those who frequently carry a firearm outside the home are often full-time employed men. Although over two-thirds of rural residents own a firearm, rural residents typically own firearms for multiple purposes (e.g., hunting, livestock and property protection) (Igielnik, 2017). Black men in Oklahoma are most often threatened with a firearm, aligning with their disproportionately high firearm-related death rates in the state (Centers for Disease Control and Prevention, 2022b) and national patterns (Kaufman et al., 2024). Contrary to common findings, (Lu et al., 2024; Peitzmeier et al., 2022) most Oklahomans threatened with a firearm report full-time employment; however, since the survey asked about lifetime exposure to being threatened by a firearm, their employment status at the time of victimization is unknown.

Nearly half of firearm owners have never received formal safety training or a concealed-carry permit. Similarly, almost half of Oklahomans who carried a firearm outside their home in the past year also lack formal safety training, and over half have never obtained a concealed-carry permit. Oklahoma's permissive firearm policies, including permitless carry (Self-Defense Act Unit, 2024), may influence perceptions about the need for safety training or permits. Additionally, whether firearm training effectively communicates safe storage practices or educates firearm owners on strategies to prevent unauthorized firearm access at home or in public remains unclear.

The regional sample has both strengths and limitations. Participants represented 54.5% of Oklahoma counties, providing broad geographic reach for a community-based convenience sample; however, some areas may still have been underrepresented. Although this sample provides unique data on firearm behaviors and related experiences, it was not designed to be representative of the Oklahoma adult population and does not fully reflect 2024 Oklahoma census data across all racial groups (e.g., a higher percentage of non-Hispanic Black participants). Accordingly, the nonrepresentative sample limits direct comparisons with national estimates, and small subgroup sizes may reduce statistical power and precision; both should be interpreted cautiously. Lastly, the cross-sectional nature of the data precludes temporal inferences.

## 5 Conclusions

The 2024 Oklahoma Firearm Survey (OFS) provides critical insight into firearm ownership, storage, and exposure to firearm-related violence in Oklahoma. Many Oklahomans own firearms, often store them unsafely, and carry firearms outside the home. Moreover, Black men face the highest exposure to firearm violence. Future research should focus on large, representative samples to better understand statewide disparities across these firearm-related behaviors and experiences. Given Oklahoma's permissive firearm policies and high rates of unsafe storage, these findings highlight the need for targeted interventions to promote safe firearm behaviors that will ideally reduce firearm-related violence in the state.

## CRediT authorship contribution statement

**Hillary McGuire:** Writing – review & editing, Writing – original draft, Visualization, Project administration, Methodology, Investigation, Funding acquisition, Formal analysis, Data curation, Conceptualization. **Marshall K. Cheney:** Writing – review & editing, Conceptualization. **Daniel Larson:** Writing – review & editing, Conceptualization. **Hairong Song:** Writing – review & editing, Conceptualization. **Jeff R. Temple:** Writing – review & editing, Conceptualization. **Yu Lu:** Writing – review & editing, Writing – original draft, Visualization, Supervision, Project administration, Methodology, Investigation, Funding acquisition, Formal analysis, Data curation, Conceptualization.

## Ethical compliance

The Institutional Review Board of the University of Oklahoma approved this study (#16357).

## Funding

This research was supported by the Junior Faculty Summer Fellowship (PI: Lu) from the Dodge Family College of Arts and Sciences of the 10.13039/100007926University of Oklahoma, (PI: McGuire) from the 10.13039/100007926University of Oklahoma 10.13039/100006298Graduate College, Graduate Student Senate Research Award (PI: McGuire) from the 10.13039/100007926University of Oklahoma Student Government Association, and Helen Riddle Student Grant Award (PI: McGuire) from the 10.13039/100007926University of Oklahoma 10.13039/501100003921Department of Health and Exercise. The content is solely the authors' responsibility and does not necessarily represent the official views of the funders.

## Declaration of competing interest

The authors declare that they have no known competing financial interests or personal relationships that could have appeared to influence the work reported in this paper.
