# Supplementary material for: Guns in the heartland: examining firearm behaviors among Oklahoman adults
Source: Prev Med Rep. 2026 Oct 18;71:103673. doi: 10.1016/j.pmedr.2026.103673 (PMC13718665; doi:10.1016/j.pmedr.2026.103673)
Supplement: Supplementary file 1 [file mmc1.docx]

**Supplemental Materials**

| **Supplemental Table 1: Differences in firearm ownership, storage, and carrying by firearm safety training and concealed-carry permit status among adults in Oklahoma, United States, 2024** | | | | | | | | |
| --- | --- | --- | --- | --- | --- | --- | --- | --- |
|  | **Firearm safety training** | |  |  | **Concealed-carry permit** | |  |  |
|  | **Never received training** | **Ever received training** |  |  | **Never licensed** | **Ever had a license** |  |  |
|  | **N (%)** | **N (%)** | **Phi** | **χ²** | **N (%)** | **N (%)** | **Phi** | **χ²** |
| **Firearm ownership** |  |  | .433 | 109.0  (*p* <.001) |  |  | .436 | 110.4  (*p* <.001) |
| Yes | 141 (48.3) | 151 (51.7) |  |  | 185 (63.4) | 107 (36.6) |  |  |
| No | 256 (88.6) | 33 (11.4) |  |  | 282 (97.9) | 6 (2.1) |  |  |
| **Firearm Storage among Firearm Owners** |  |  | .082 | 1.96  (*p* =.161) |  |  | .042 | .515  (*p* =.473) |
| Safe | 10 (35.7) | 18 (64.3) |  |  | 16 (57.1) | 12 (42.9) |  |  |
| Unsafe | 131 (49.6) | 133 (50.4) |  |  | 169 (64.0) | 95 (36.0) |  |  |
| **Firearm carrying** |  |  | .279 | 45.2  (*p* <.001) |  |  | .378 | 83.1  (*p* <.001) |
| Yes | 85 (48.6) | 90 (51.4) |  |  | 101 (57.7) | 74 (42.3) |  |  |
| No | 312 (76.8) | 94 (23.2) |  |  | 366 (90.4) | 39 (9.6) |  |  |
| *Note.* The overall analytic sample included 630 adults; firearm storage analyses were limited to firearm owners (*n* = 319). Reported percentages are row percentages.  *P* values were obtained using Pearson chi-square tests or Fisher’s exact tests, as appropriate. | | | | | | | | |
